# Rapid Shallow Breathing Index as a Predictor of Extubation Outcomes After Pediatric Cardiac Surgeries

**DOI:** 10.3390/children13040503

**Published:** 2026-04-02

**Authors:** Mustafa Saad El Masri, Wajih Nasr, Marianne N. Majdalani, Jihane Moukhaiber

**Affiliations:** 1 Faculty of Medicine, American University of Beirut, Beirut P.O. Box 11-0236, Lebanon; mms156@mail.aub.edu (M.S.E.M.); wgn01@mail.aub.edu (W.N.); 2 Pediatric Critical Care, Department of Pediatrics and Adolescent Medicine, American University of Beirut Medical Center, Beirut P.O. Box 11-0236, Lebanon; mn40@aub.edu.lb

**Keywords:** RSBI, cardiac surgery, pediatric, extubation failure

## Abstract

**Highlights:**

**What are the main findings?**

- The RSBI measured just before extubation was a strong predictor of the extubation outcome in children after cardiac surgery; patients who failed extubation had significantly higher RSBI values, and an RSBI cutoff ≥ 4.62 breaths/min/mL/kg showed strong discrimination (AUC 0.974).
- The RSBI was the only independent predictor of extubation failure on multivariable analysis reflecting the integrative nature of the RSBI as a real-time physiologic marker.

**What are the implications of the main finding?**

- The RSBI can be incorporated into spontaneous breathing trial assessments as a practical bedside tool to improve extubation readiness evaluation in pediatric cardiac ICU patients.
- An RSBI below 4.62 provides strong reassurance for safe ventilator liberation, whereas higher values should prompt a closer reassessment of reversible risk factors and more cautious extubation planning.

**Abstract:**

**Background/Objectives:** Determining the optimal timing for the discontinuation of mechanical ventilation (MV) in pediatric patients following cardiac surgery remains challenging. Both delayed and premature extubation increase the risk of complications. The rapid shallow breathing index (RSBI) is widely used, but its role and optimal cutoff in pediatric cardiac populations remain uncertain. This study aimed to determine a clinically useful RSBI cutoff for predicting extubation readiness in children after cardiac surgery. **Methods:** We conducted a prospective single-center observational cohort study including children younger than 18 years who required postoperative MV after cardiac surgery and were admitted to the Pediatric Intensive Care Unit (PICU) between July 2020 and June 2024. The RSBI was measured one minute prior to extubation during a spontaneous breathing trial (SBT). Extubation failure was defined as the need for reintubation within 48 h. **Results:** A total of 247 patients were enrolled, with 13 (5.3%) experiencing extubation failure. Patients who failed extubation had significantly higher RSBI values compared with those successfully extubated (median 4.97 vs. 3.76; *p* < 0.001). An RSBI cutoff ≥ 4.62 breaths/min/mL/kg provided a sensitivity of 84.6%, specificity of 94.0%, positive predictive value (PPV) of 44%, and negative predictive value (NPV) of 99.1%. The RSBI was the only independent predictor of extubation failure in multivariable analysis (*p* = 0.014). **Conclusions:** The RSBI is a simple and reliable physiological marker for assessing extubation readiness in pediatric patients after cardiac surgery. An RSBI threshold of ≥4.62 breaths/min/mL/kg identifies patients at increased risk of extubation failure. Larger, multicenter studies will be important to validate our results.

## 1. Introduction

Mechanical ventilation (MV) remains a fundamental component of care for critically ill patients in intensive care units (ICUs) [1]. However, determining the optimal timing for discontinuation of MV remains a complex clinical challenge. Unnecessary delays had been associated with a range of complications, including diaphragmatic weakness and atrophy, delirium and cognitive dysfunction, ventilator-associated infections, ventilator-induced lung injury (VILI), airway trauma from the endotracheal tube (ETT), and critical illness-related neuropathy or myopathy [1,2,3,4,5]. Some of these adverse effects are directly related to invasive respiratory support, while others result from prolonged exposure to sedatives, opioids, neuromuscular blocking agents, and their cumulative dosing [6,7]. Conversely, premature extubation carries its own risks, such as respiratory muscle fatigue, impaired gas exchange, loss of airway protection, and increased stress on both the respiratory and cardiovascular systems, often resulting in reintubation and worse clinical outcomes [8,9,10]. Thus, extubation represents a delicate balance between avoiding unnecessary prolongation of support and preventing failure of early liberation.

This balance is particularly challenging in pediatric patients undergoing cardiac surgery, in whom respiratory and cardiovascular systems are closely interdependent. Following cardiopulmonary bypass (CPB), children frequently experience hemodynamic instability and a systemic inflammatory response that can promote fluid overload, pulmonary edema, pleural effusions, and elevated pulmonary vascular resistance [11,12,13]. Postoperative respiratory recovery may be further compromised by generalized muscle weakness and diaphragmatic fatigue, both of which are common in this population [14,15]. In addition, cardiac-specific factors—arising across the preoperative, intraoperative, and postoperative periods—can significantly influence extubation outcomes. Although these conditions primarily affect the heart, their downstream cardiopulmonary effects often determine readiness for ventilator liberation. Such factors include residual cardiac lesions, prolonged CPB or aortic cross-clamp times, intraoperative cardiac arrest, arrhythmias, myocardial stunning, and pericardial effusion, among others [16,17,18]. These factors, often acting simultaneously, make the assessment of extubation readiness in pediatric cardiac patients uniquely complex.

Current International clinical practice guidelines for pediatric ventilator liberation advocate the use of standardized extubation readiness testing (ERT), commonly incorporating spontaneous breathing trials (SBTs) [19,20]. However, these recommendations are largely derived from studies involving heterogeneous pediatric ICU populations, with limited representation from cardiac intensive care units (CICUs). In practice, adherence to SBTs in pediatric CICUs remains inconsistent; Sher et al. reported that only 43.7% of providers routinely performed SBTs in this setting [21], and decision-making often relies on a combination of clinical judgment and nonspecific physiologic indicators. As a result, there is a need for simple, objective, and reproducible bedside tools that can complement existing strategies and improve the precision of extubation readiness assessment in this population.

The rapid shallow breathing index (RSBI), defined as the ratio of respiratory rate (RR) to tidal volume (TV) adjusted for body weight (breaths/min/mL/kg), is one of the most widely studied predictors of successful liberation from MV. It reflects the relationship between respiratory demand and ventilatory capacity at the time of assessment. Despite its established role in adult critical care, its utility in pediatric critical care remains uncertain [22,23,24]. Although current guidelines recommend measuring the RSBI during SBTs, no pediatric-specific cutoff value has been established [19,20]. Moreover, only a limited number of studies have examined its utility in children post congenital cardiac surgery. Johnston et al. found no association between RSBI and extubation failure [25], whereas Munshi et al. observed higher RSBI values among children who failed extubation, though they were unable to define a reliable threshold due to small sample size [26].

Given these limitations, the primary objective of our study was to identify a clinically meaningful RSBI cutoff that could reliably predict safe extubation in pediatric cardiac patients. We hypothesized that incorporating an evidence-based RSBI threshold into SBTs may enhance clinical decision-making and improve the safety and efficiency of ventilator liberation in this high-risk population.

## 2. Materials and Methods

### 2.1. Study Design and Data Source

We performed a prospective, single-center observational cohort study involving children younger than 18 years of age who were admitted to the PICU following cardiac surgery at AUBMC between 1 July 2020, and 30 June 2024. The study was approved by the Institutional Review Board under the title “Determination of Rapid Shallow Breathing Index as a Predictor of Extubation Readiness in the Pediatric Intensive Care Unit” (BIO-2019-0335, amendment approved on 4 June 2020). Written informed parental consent was obtained for all participants, and the study was conducted in accordance with the Declaration of Helsinki.

Demographic and clinical data were prospectively collected from the electronic medical record system (EPIC), as well as directly from ventilator parameters, following caregiver consent. All patients were monitored for a minimum of 48 h after extubation. In cases of extubation failure, follow-up continued until at least 48 h after successful re-extubation.

Data collection was organized into three main domains. The first included demographic information such as date of birth, sex, and weight. The second captured clinical characteristics at admission, including primary diagnosis, Risk Adjustment for Congenital Heart Surgery-1 (RACHS-1) score and associated comorbidities. The third focused on PICU course and management, documenting the date and time of PICU admission, intubation, total duration of mechanical ventilation, extubation timing and outcome, and documented causes of extubation failure. Details regarding analgesic, sedative, and neuromuscular blocking agents—including cumulative doses—were recorded. Respiratory parameters, including RR, TV, and calculated RSBI, were measured one minute prior to extubation, along with the type of respiratory support provided following extubation.

### 2.2. Study Population

The study included intubated pediatric patients following cardiac surgery, younger than 18 years of age who were admitted to the PICU at AUBMC and judged by the attending intensivist to be ready for extubation. Children who experienced extubation failure and required re-intubation within 48 h were followed until they remained successfully extubated for at least 48 h after re-extubation.

Patients were excluded if they met any of the following criteria: age 18 years or older, neonatal age group, non-cardiac patients, presence of an underlying neuromuscular disorder, requirement for tracheostomy after re-intubation following a failed first extubation, or transfer to another healthcare facility either before extubation or within 48 h after extubation.

### 2.3. Outcome and Predictor Variables

The primary outcome of this study was to determine a clinically useful RSBI cutoff that could help predict extubation readiness in pediatric cardiac patients. The RSBI was calculated as the ratio of RR to TV adjusted for body weight, and is expressed in breaths/min/mL/kg.

### 2.4. Recruitment Strategy and Sampling Technique

The study was conducted in the PICU at AUBMC, a tertiary referral center in Lebanon. The PICU is a closed, 10-bed unit that admits cardiac patients post operatively. The unit is staffed by three pediatric intensivists who follow a unified and standardized approach to ventilator weaning and extubation.

All intubated pediatric cardiac patients were assessed daily for readiness to wean from MV. Extubation readiness was determined by the attending intensivist based on standardized institutional criteria (detailed in Supplementary Material S1).

Once deemed ready, patients underwent an SBT using a spontaneous ventilation mode with pressure support ranging from 6 to 12 cmH_2_O, adjusted according to the ETT size (detailed in Supplementary Material S2). PEEP was set between 4 and 5 cmH_2_O, and FiO_2_ was tailored to the underlying disease. Patients who tolerated the SBT for one hour without clinical or hemodynamic compromise and were afebrile were considered eligible for extubation. At that time, RR (breaths per minute) and TV (mL/kg) were recorded directly from the ventilator one minute prior to extubation. The RSBI was then calculated as the ratio of RR to TV.

The extubation protocol described above represents the standard of care in the unit and was applied to all intubated pediatric patients, irrespective of participation in the study. Additional clinical and demographic data were extracted from EPIC. Following extubation, patients were monitored for 48 h to assess extubation outcomes. In cases where re-intubation was required within this period, follow-up continued until the patient remained successfully extubated for at least 48 h after re-extubation.

### 2.5. Statistical Analysis

Reported rates of extubation failure vary widely across institutions, reaching as high as 30% in some settings [27]. Based on our center’s experience over the past five years, we estimated an expected extubation failure rate of 10% and used this value to calculate the required sample size for the study. Assuming a 95% confidence interval and a margin of error of 0.04, the minimum sample size was calculated as 217 patients using Cochran’s formula:

$$N=\;\frac{Z^{2}.p.(1-p)}{d^{2}}$$

where N represents the required sample size, Z the Z-score corresponding to a 95% confidence level, p the anticipated proportion, and *d* the desired margin of error.

To strengthen the statistical power of our analysis, we ultimately enrolled 247 patients over a four-year period. Continuous variables were summarized using medians and interquartile ranges (IQRs), as their distributions were non-normal. Categorical variables were described using frequencies and percentages. Body mass index (BMI) was categorized according to World Health Organization BMI-for-age percentiles into four groups (≤15th percentile, <50th percentile, <85th percentile, and ≥85th percentile) [28].

Descriptive statistical analyses were conducted to summarize the demographic and clinical characteristics of the intubated pediatric cardiac population. Categorical variables were compared using the chi-square or Fisher’s exact test, as appropriate, while continuous variables were analyzed using the independent samples *t*-test. Variables with a *p*-value ≤ 0.20 in univariable analysis were entered into a multivariable regression model to identify independent predictors of extubation failure.

Receiver operating characteristic (ROC) curve analysis was subsequently performed to determine the optimal RSBI cutoff and evaluate its diagnostic performance. Sensitivity, specificity, PPV, and NPV were calculated to assess the predictive accuracy of the RSBI for extubation failure. A *p*-value of less than 0.05 was considered statistically significant.

## 3. Results

A total of 247 pediatric patients who underwent cardiac surgery and required postoperative MV were enrolled over the four-year study period (Table 1). The median age of the cohort was 2.0 years. Overall, 13 patients (5.3%) experienced extubation failure, while 234 (94.7%) remained successfully extubated.

BMI percentile was significantly associated with extubation failure (*p* = 0.001), with a higher proportion of reintubated patients falling at or below the 15th percentile.

The distribution of underlying cardiac diagnoses differed significantly between patients with successful extubation and those who failed extubation (*p* < 0.001). Extubation failure occurred more frequently among patients with single-ventricle palliation, including Bidirectional Glenn (BDG) and Fontan procedures, as well as those with atrioventricular canal (AVC) defects and Blalock–Taussig (BT) shunt physiology.

Surgical complexity, as assessed by the RACHS-1 classification, was strongly associated with extubation outcome (*p* < 0.001). Patients with RACHS-1 category ≥3 accounted for the majority of extubation failures, whereas those with RACHS-1 category 2 were predominantly successfully extubated.

Peri-extubation characteristics were shown in Table 2. Patients who experienced extubation failure had a significantly higher RSBI measured immediately prior to extubation compared with those who were successfully extubated (median 4.97 vs. 3.76, *p* < 0.001). They also required longer durations of MV (median 32 h vs. 17 h, *p* = 0.032).

Use of neuromuscular blocking agents was significantly more common in patients who failed extubation (23.1% vs. 2.6%, *p* = 0.008). Although higher Morphine Milligram Equivalent (MME) doses were observed in the extubation failure group, this difference did not reach statistical significance. Sedation strategy (midazolam alone versus combination therapy) was also not significantly associated with extubation outcome.

All patients who failed extubation required non-invasive positive pressure ventilation (NIPPV) immediately following extubation, compared with approximately half of successfully extubated patients (*p* = 0.001).

In multivariable regression analysis, the RSBI was the only variable independently associated with extubation failure (*p* = 0.014).

The most common cause of reintubation was respiratory infection (53.8%), followed by atelectasis (23.1%) (Table 3).

The diagnostic performance of the RSBI was checked using ROC curve analysis (see Supplementary Material S3). It showed a strong discriminatory ability of the RSBI for predicting extubation failure, with an AUC of 0.974 (95% CI: 0.947–1.000, *p* < 0.001). An RSBI cutoff value of ≥4.62 breaths/min/mL/kg was identified as the optimal threshold, yielding a sensitivity of 84.6%, specificity of 94.0%, NPV of 99.1%, and PPV of 44%.

**Figure 1:**
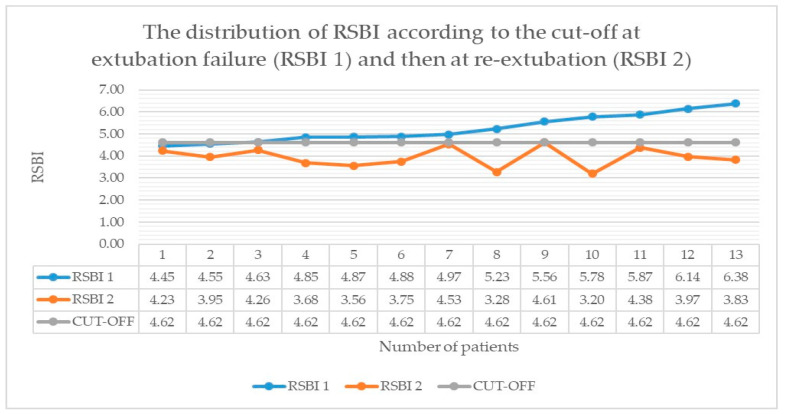
The distribution of RSBI according to the cut-off at extubation failure (RSBI 1) and then at re-extubation (RSBI 2).

In patients who experienced extubation failure, the RSBI (RSBI-2) was reassessed at the time of successful re-extubation. Figure 1 demonstrates that RSBI-2 values in all 13 patients remained below the established cutoff.

## 4. Discussion

In this prospective cohort of pediatric patients undergoing cardiac surgery, we demonstrated that the RSBI measured immediately prior to extubation is a strong predictor of extubation outcome. To our knowledge, this study represents one of the largest prospective evaluations of the RSBI in pediatric patients following cardiac surgery and is among the first to propose a clinically actionable RSBI cutoff derived from a standardized extubation protocol.

The RSBI is a real-time physiologic marker that captures the combined effects of respiratory muscle strength, lung mechanics, neurologic status, and metabolic demand at the moment of extubation. Its relevance in this population in particular, can be better understood in the context of post-CPB pathophysiology. Following cardiac surgery, systemic inflammation and capillary leak contribute to pulmonary edema and reduced lung compliance, leading to lower TVs [11,12,13]. Concurrently, diaphragmatic dysfunction and respiratory muscle fatigue—well described in patients receiving MV—can impair effective ventilation and promote compensatory increases in RR [14,15]. In addition, residual cardiac lesions, shunt physiology, or postoperative myocardial dysfunction may increase metabolic demand and ventilatory requirements, further stressing the balance between respiratory load and capacity [16,18]. By integrating RR and TV into a single index, RSBI reflects the net effect of these interacting processes at the time of extubation.

However, the physiologic framework and integrative property mentioned above does not fully explain why the RSBI emerged as the only independent predictor of extubation failure in multivariable regression analysis. Although similar observations have been reported in pediatric and adult studies, where dynamic respiratory indices measured during SBTs outperform baseline or perioperative characteristics in adjusted analyses [8,19,22,24], this result should be interpreted with caution in our study. The limited number of outcome events raises the possibility of overfitting and restricts the robustness of multivariable modeling.

In adults, an RSBI threshold of approximately 105 breaths/min/L is widely used [8,9]. However, this value is not directly applicable to children due to fundamental differences in respiratory physiology, including higher RR and smaller TV. Pediatric studies have reported heterogenous RSBI cutoff values for predicting successful extubation, with thresholds ranging from approximately 6.7 breaths/min/mL/kg [22,29], to around 8 breaths/min/mL/kg [30], and up to 11 breaths/min/mL/kg [31]. The comparatively lower threshold identified in our study (4.62 breaths/min/mL/kg) may be explained by the relatively homogeneous population of physiologically optimized postoperative cardiac patients, in contrast to prior studies that included heterogeneous PICU populations with diverse underlying conditions. Although the RSBI demonstrated strong discriminative performance (AUC 0.974; 95% CI 0.947–1.000), the small number of events may have introduced optimism bias and led to overestimation of model performance.

While the RSBI measures real-time respiratory load/capacity, BMI reflects baseline reserve. Multiple studies supported the association between lower BMI percentile and extubation failure. Poor growth and low lean body mass have been linked to diaphragm weakness, increased respiratory workload, reduced cough efficacy, delayed weaning and increased morbidity [17,23,24,32,33].

In our cohort, all patients who experienced extubation failure received NIPPV immediately after extubation, highlighting that clinicians appropriately identified these patients as high risk and escalated support early. This finding should be interpreted cautiously because non-invasive respiratory support (NRS) use is often influenced by confounding by indication: clinicians preferentially deploy NRS in patients perceived to have marginal reserve, which can make it appear associated with worse outcomes in observational data even when it is beneficial. Recent pediatric evidence supports the role of post-extubation NRS as a preventive strategy in children at risk for extubation failure [34,35,36,37,38].

Extubation failure in our cohort occurred more frequently in patients with higher surgical complexity (RACHS-1 ≥ 3), including those undergoing single-ventricle palliation such as BDG and Fontan procedures, as well as AVC repairs. In contrast, extubation failure was rare or absent in several biventricular repairs such as isolated ventricular septal defect (VSD) and tetralogy of Fallot (TOF) repair. This distribution is consistent with the multicenter findings reported by Byrnes et al. [32], where procedures involving cavopulmonary physiology, AVC repair, and higher RACHS-1 categories exhibited higher rates of both early and late extubation failure, whereas isolated biventricular repairs demonstrated lower failure rates. These similarities reinforce the concept that extubation failure is largely driven by underlying cardiopulmonary physiology and surgical complexity, rather than by center-specific practices alone.

Although the absolute proportions in our single-center subgroups appear higher for some procedures (e.g., Fontan, BDG), interpretation must be cautious due to small sample sizes. Nonetheless, the overall pattern aligns well with large registry data.

Patients who failed extubation in our study required significantly longer durations of MV and were more likely to have received neuromuscular blocking agents. Similar associations have been reported in prior studies, where prolonged ventilation often reflects greater illness severity, diaphragmatic dysfunction, pleural effusions, or postoperative complications rather than failure of weaning assessment alone [10,16,17,32].

International pediatric ventilator liberation guidelines emphasize neurologic readiness and airway protection as critical components of extubation readiness [19,20]. By allowing additional time for sedative washout before attempting extubation, our approach may attenuate the impact of sedation on extubation outcomes. This may partially explain why sedation did not emerge as an independent risk factor in our cohort, despite its known association with prolonged ventilation and delirium in other settings [2,6,7].

The strongest clinical contribution of our study lies in the very high NPV of the RSBI. In a population with a relatively low extubation failure rate, a tool that reliably identifies patients at low risk of failure is particularly valuable. An RSBI below 4.62 breaths/min/mL/kg can provide reassurance to clinicians when other clinical readiness criteria are satisfied, while elevated values should prompt reassessment of reversible factors rather than serve as an absolute contraindication to extubation.

## 5. Limitations

This study has several limitations. Although it was conducted prospectively in a tertiary care center with access to advanced resources—thereby strengthening its internal validity—it remains a single-center study. As such, the findings may not be fully generalizable to other institutions with different clinical protocols, resource availability, or patient populations, including other hospitals across Lebanon and beyond.

The high discriminatory performance of the RSBI observed in this study should be interpreted with caution. Only 13 extubation failure events occurred, which introduces the possibility of small-event inflation bias and potential overestimation of model performance. In addition, we did not perform internal validation techniques, such as bootstrapping, to assess model stability. Therefore, the proposed RSBI cutoff and its diagnostic performance should be considered preliminary and require validation in larger, multicenter cohorts before broader clinical application.

The interpretation of the multivariable analysis is similarly limited by the small number of outcome events. According to established events-per-variable recommendations, the number of covariates that can be reliably included in regression models is restricted, increasing the risk of overfitting. Consequently, the identification of the RSBI as an independent predictor should be considered exploratory and hypothesis-generating rather than definitive. Larger studies with sufficient event numbers are needed to confirm the stability and independence of this association.

Finally, the RSBI was measured at a single time point immediately prior to extubation, and dynamic changes during the spontaneous breathing trial were not assessed. Although patients were continuously monitored clinically throughout the trial, serial RSBI measurements may provide additional insight into evolving respiratory muscle fatigue and could further refine extubation readiness assessment. This represents an important area for future investigation.

## 6. Conclusions

The RSBI is a simple, reproducible, and physiologically meaningful tool that can enhance extubation readiness assessments in pediatric patients following cardiac surgery. An RSBI cutoff of ≥4.62 breaths/min/mL/kg identifies patients at increased risk of extubation failure, while values below this threshold provide strong reassurance for safe ventilator liberation. Larger, multicenter studies are needed to validate our results.

## Figures and Tables

**Table 1:** Demographics and characteristics of intubated cardiac patients.

	Reintubation			
	No	Yes	Total	
	Count (%) Median (IQR)	Count (%) Median (IQR)	Count (%) Median (IQR)	*p*-Value
Age (years)	2.00 (0.85–5.30)	1.58 (0.35–7.00)	2.00 (0.83–5.30)	0.397
Male	122 (52.1%)	3 (23.1%)	125 (50.6%)	
Female	112 (47.9%)	10 (76.9%)	122 (49.4%)	
BMI percentile				0.001
15th percentile and below	62 (26.5%)	9 (69.2%)	71 (28.7%)	
<50th percentile	74 (31.6%)	1 (7.7%)	75 (30.4%)	
<85th percentile	60 (25.6%)	0 (0%)	60 (24.3%)	
85th percentile and above	38 (16.2%)	3 (23.1%)	41 (16.6%)	
Diagnosis				<0.001
VSD	60 (25.6%)	0 (0%)	60 (24.3%)	
ASD	28 (12%)	2 (15.4%)	30 (12.1%)	
TOF	28 (12%)	0 (0%)	28 (11.3%)	
Coarctation of the aorta	24 (10.3%)	0 (0%)	24 (9.7%)	
BDG	20 (8.5%)	3(23.1%)	23 (9.3%)	
RV-PA conduit	22 (9.4%)	0 (0%)	22 (8.9%)	
AVC	18 (7.7%)	2 (15.4%)	20 (8.1%)	
Fontan	12 (5.1%)	3 (23.1%)	15 (6.1%)	
Mitral Valve	8(3.4%)	1 (7.1%)	9 (3.6%)	
Pulmonary Valve	6 (2.6%)	0 (0%)	6 (2.4%)	
TAPVR	6 (2.6%)	0 (0%)	6 (2.4%)	
BT shunt	0 (0%)	2 (15.4%)	2 (0.8%)	
Tricuspid Valve Lesion	2 (0.9%)	0 (0%)	2 (0.8%)	
RACHS-1				<0.001
1	24 (10.3%)	2 (15.4%)	26 (10.5%)	
2	130 (55.6%)	1 (7.7%)	131 (53%)	
3	76 (32.5%)	8 (61.5%)	84 (34%)	
4	4 (1.7%)	0 (0%)	4 (1.6%)	
6	0 (0%)	2 (15.4%)	2 (0.8%)	
Comorbidity				0.555
No	156 (66.7%)	10 (76.9%)	166 (67.2%)	
Yes	78 (33.3%)	3 (23.1%)	81 (32.8%)	

(ASD: atrial septal defect; AVC: atrioventricular canal; BDG: Bidirectional Glenn; BMI: body mass index; BT: Blalock–Taussig; IQR: interquartile range; RV-PA: right ventricular to pulmonary artery; TAPVR: total anomalous pulmonary venous return; TOF: tetralogy of Fallot; VSD: ventricular septal defect).

**Table 2:** Peri-extubation characteristics.

		Reintubation			*p*-Value
		No	Yes	Total	
RSBI 1		3.76 (3.29–4.33)	4.97 (4.85–5.78)	3.82 (3.33–4.37)	<0.001
Ventilation hours		17 (12–30)	32 (21–107)	17 (13–30)	0.032
MME/kg		2.52 (1.56–4.27)	5.65 (3.65–18)	2.57 (1.57–5.32)	0.096
Sedation (Count (%))	Midazolam	214 (91.5%)	10 (76.9%)	224 (90.7%)	0.109
	Combination	20 (8.5%)	3 (23.1%)	23 (9.3%)	
Muscle Relaxant (Count (%))	No	228 (97.4%)	10 (76.9%)	238 (96.4%)	0.008
	Yes	6 (2.6%)	3 (23.1%)	9 (3.6%)	
NIPPV (Count (%))	No	112 (47.9%)	0 (0%)	112 (45.3%)	0.001
	Yes	122 (52.1%)	13(100%)	135 (54.7%)	

(MME: Morphine Milligram Equivalent; NIPPV: non-invasive positive pressure ventilation; RSBI: Rapid Shallow Breathing Index).

**Table 3:** Causes of reintubation.

Causes of Reintubation	Frequency (%)
Infection	7 (53.8%)
Atelectasis	3 (23.1%)
Neurologic cause	1 (7.7%)
Cardiac failure post-extubation	1 (7.7%)
Vomiting/Aspiration during extubation	1 (7.7%)
Total	13 (100%)

## Data Availability

The raw data supporting the conclusions of this article will be made available by the authors on request.

## Supplementary Materials

The following supporting information can be downloaded at: https://www.mdpi.com/article/10.3390/children13040503/s1, File S1: Extubation Readiness Criteria; File S2: Pressure Support range according to endotracheal tube size. File S3: ROC Curve of RSBI for Predicting Extubation Failure.

## Abbreviations

The following abbreviations are used in this manuscript:

ASD	Atrial septal defect
AVC	Atrioventricular canal
BDG	Bidirectional Glenn
BMI	Body mass index
BT	Blalock–Taussig
CICU	Cardiac Intensive Care Unit
CPB	Cardiopulmonary bypass
ERT	Extubation readiness testing
ETT	Endotracheal tube
ICU	Intensive Care Unit
IQR	Interquartile range
MV	Mechanical ventilation
NIPPV	Non-invasive positive pressure ventilation
NPV	Negative predictive value
NRS	Non-invasive respiratory support
PICU	Pediatric Intensive Care Unit
PPV	Positive predictive value
RACHS-1	Risk Adjustment for Congenital Heart Surgery-1
ROC	Receiver Operating Characteristic
RR	Respiratory rate
RSBI	Rapid Shallow Breathing Index
RV-PA	Right ventricular to Pulmonary artery
SBT	Spontaneous Breathing Trial
TAPVR	Total anomalous pulmonary venous return
TOF	Tetralogy of Fallot
TV	Tidal volume
VILI	Ventilator induced lung injury
VSD	Ventricular septal defect
