# Supplementary material for: Rapid Shallow Breathing Index as a Predictor of Extubation Outcomes After Pediatric Cardiac Surgeries
Source: Children (Basel). 2026 Apr 2;13(4):503. doi: 10.3390/children13040503 (PMC13114925; doi:10.3390/children13040503)

Supplementary material

Supplementary Material S1:

Extubation Readiness Criteria included:

- adequate neurological status (presence of cough and gag reflexes and a State Behavioral Scale score between −1 and 0),
- stable pulmonary function (ability to maintain airway patency, effective cough with minimal secretions, satisfactory oxygenation and ventilation with PEEP ≤5 cmH_2O and FiO_2 ≤60%, normal minute ventilation, a positive leak test, improving or normal chest radiography, and acceptable arterial blood gas values),
- and cardiovascular stability (age-appropriate heart rate, normal blood pressure, good peripheral perfusion, normal capillary refill time, and adequate urine output). In addition, good cardiac function by cardiac ultrasound.

Supplementary Material S2:

Title: Pressure Support range according to endotracheal tube size.

**ETT size**	**Pressure Support range**
≤ 3.5 mm	10–12 cmH_2O
4.0–4.5 mm	8–10 cmH_2O
≥ 5.0 mm	6–8 cmH_2O

Supplementary Material S3:

Title: ROC Curve of RSBI for Predicting Extubation Failure

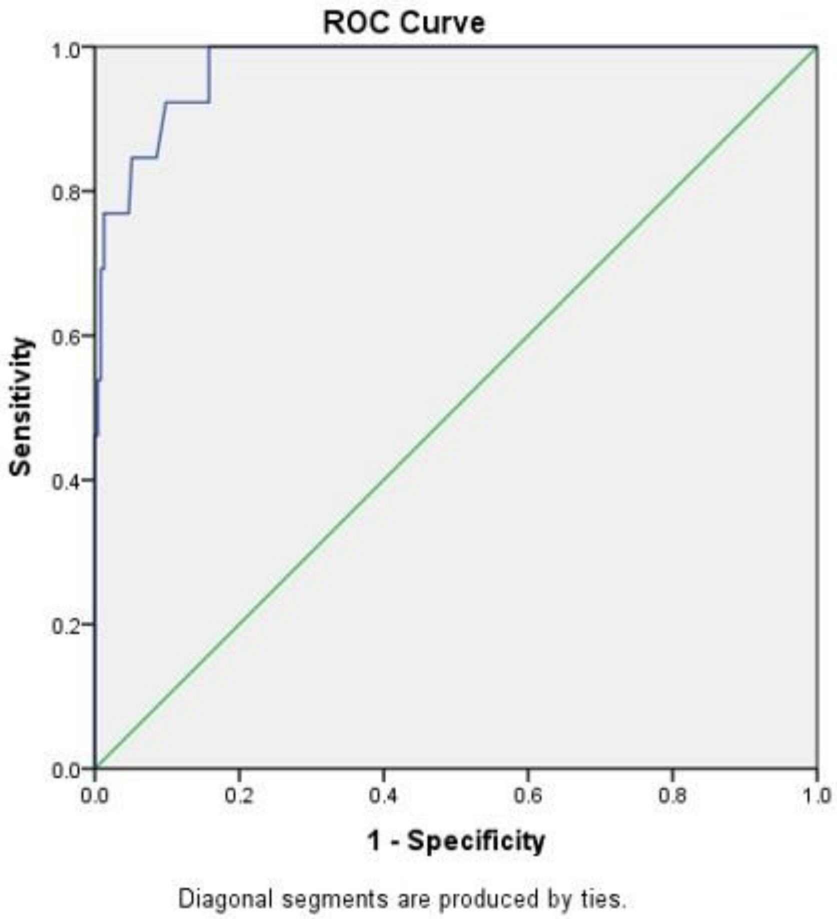

Supplement: Supplementary file 1 [file children-13-00503-s001.zip › children-4217835-supplementary.pdf]
